# Supplementary material for: Cytotoxic and Transcriptomic Effects in Avian Hepatocytes Exposed to a Complex Mixture from Air Samples, and Their Relation to the Organic Flame Retardant Signature
Source: Toxics. 2021 Nov 30;9(12):324. doi: 10.3390/toxics9120324 (PMC8704741; doi:10.3390/toxics9120324)
Supplement: Supplementary file 1 [file toxics-09-00324-s001.zip › toxics-1403287-supplementary.pdf]

toxics MDPI

Supplementary Materials: Cytotoxic and Transcriptomic Effects in Avian Hepatocytes Exposed to a Complex Mixture from Air Samples, and Their Relation to the Organic Flame Retardant Signature

Kelsey Ha, Pu Xia, Doug Crump, Amandeep Saini, Tom Harner and Jason O'Brien

Table S1. List of sampling location information including sampling dates for period 2 (start and retrieval), exposure time, total population, density of the cities, gross domestic product (GDP) and human development index (HDI). These data were provided by Saini et al. 2020 [1].

Location	Latitude	Longitude	Start Date	Retrieval Date	Exposure Time (days)	Population[1] (million)	Pop Density[1] (per sq km)	GDP[2] (billion USD) PPP Adjusted	HDI[3]
Bangkok, Thailand	13.7235	100.5216	28-Mar-18	27-Jun-18	91	8.3/14.6	5300	306.8	0.755
Beijing, China	39.9042	116.4074	02-Jul-18	01-Oct-18	91	21.5/24	1300	506	0.887
Cairo, Egypt	30.0141	31.4860	02-Jul-18	02-Oct-18	92	12/20.5	19,376	102.2	0.743
São Paulo, Brazil	–23.6183	–46.6355	02-Jul-18	02-Oct-18	92	12.2/21.5	7216	430.5	0.826
Mexico city, Mexico	19.2465	–99.1013	02-Jul-18	02-Oct-18	92	8.9/21.2	6000	403.6	0.83
Madrid, Spain	40.4432	–3.6846	02-Jul-18	02-Oct-18	92	3.3/6.5	5400	262.3	0.925
Sydney, Australia	–33.7962	151.1449	03-Jul-18	03-Oct-18	92	5.5*	400*	223.4	0.94
Lagos, Nigeria	6.5244	3.3792	30-Jun-18	30-Sep-18	92	13/21	6871	33.6	0.652
Bogota, Colombia	4.6368	–75.0834	29-Jun-18	03-Oct-18	96	8.1/11.5	4310	159.9	0.794
Warsaw, Poland	52.2216	21.0073	03-Jul-18	28-Sep-18	87	1.8	3372	141.1	0.865
Tokyo, Japan	35.6895	139.6917	09-Jul-18	01-Oct-18	84	9.3/13.5	6158	1617	0.936
Toronto, Canada	43.6590	–79.3956	03-Jul-18	03-Oct-18	92	2.8/5.9	4150	276.3	0.926
Istanbul, Turkey	41.0082	28.9784	12-Apr-18	12-Jul-18	91	15	2523	348.7	0.812
London, UK	51.4960	–0.1270	12-Jul-18	08-Oct-18	88	8.7/14	5600	835.7	0.965
New York, USA	40.7301	–73.9989	01-Jul-18	13-Nov-18	135	8.6	10,715	1403	0.946
Kolkata, India	22.5726	88.3639	08-Jul-18	22-Oct-18	106	4.6/14.7	2,4000	60.4	0.637
Santiago, Chile	–34.5581	–58.4867	01-Mar-19	13-Jun-19	104	6.5	9821	171.4	0.874
New Delhi, India	28.5897	77.2257	25-Feb-19	17-May-19	81	16.3/29.4	11,300	293.6	0.744
Buenos Aires, Argentina	–33.4378	70.6504	17-Jan-19	28-Feb-19	42	2.89/13.5	13,680	315.9	0.885

[1]: Total population and population density data: http://worldpopulationreview.com/world-cities/. The cities for which two values of total population is given, the first value represents the population of urban agglomeration (main urban core) and the 2nd value includes the population of the metro area that includes densely populated urban core as well as surrounding territories.[2]: GDP data: GDP by metropolitan area- https://www.bea.gov/data/gdp/gdp-metropolitan-area. Note that GDP data given here is purchasing power parity (PPP) adjusted for which rates of a currency conversion are applied to eliminate the differences in price levels between different countries' currencies.[3]: HDI: Unites Nations Development Programme - Human Development Index Trends. http://hdr.undp.org/en.* http://www.population.net.au/sydney-population/.

Table S2. List of target analytes, standards and their abbreviations

Chemical	Abbreviation (alternate abbr.)
2,2',4-Tribromodiphenyl ether	BDE-17
2,4,4'-Tribromodiphenyl ether	BDE-28
2,2',4,4'-Tetrabromodiphenyl ether	BDE-47

2,3',4,4'-Tetrabromodiphenyl ether	BDE-66
2,3',4',6-Tetrabromodiphenyl ether	BDE-71
2,2',3,4,4'-Pentabromodiphenyl ether	BDE-85
2,2',4,4',5-Pentabromodiphenyl ether	BDE-99
2,2',4,4',6-pentabromodiphenyl ether	BDE-100
2,2',3,4,4',5'-hexabromodiphenyl ether	BDE-138
2,2',4,4',5,5'-hexabromodiphenyl ether	BDE-153
2,2',4,4',5,6'-hexabromodiphenyl ether	BDE-154
2,2',3,4,4',5',6-heptabromodiphenyl ether	BDE-183
2,3,3',4,4',5,6-heptabromodiphenyl ether	BDE-190
Decabromodiphenyl ether	BDE-209
Ally-2,4,6-tribromophenyl ether	TBP-AE (ATE)
Beta tetrabromoethylcyclohexane	β-DBE-DBCH (TBECH)
Pentabromobenzene	PBBZ
Pentabromoethylbenzene	PBEB
Pentabromotoulene	PBT
2-bromoallyl-2,4,6-tribromophenyl ether	BATE
Beta 1,2,5,6 tetrabromocyclooctane	β-TBCO
2,3-dibromopropyl-2,4,6-tribromophenyl ether	TBP-DBPE (DPTE)
Hexabromobenzene	HBB
Dechlorane 602	Dec-602
2-ethylhexyl-2,3,4,5-tetrabromobenzoate	EH-TBB (TBB)
Dechlorane 604	Dec-604
1,2-bis(2,4,6-tribromophenoxy)ethane	BTBPE
Bis(2-ethyl-1-hexyl)tetrabromophthalate	BEHTEBP (TBPH)
Syn- and anti-Dechlorane plus	*syn*-DP, *anti*-DP
Octabromotrimethylphenylindane	OBTMPI (OBIND)
Decabromodiphenyl ethane	DBDPE
Tetrabromobisphenol-A	TBBPA
α-Hexabromocyclododecane	α-HBCDD
β-Hexabromocyclododecane	β-HBCDD
γ-Hexabromocyclododecane	γ-HBCDD
Tri-methyl phosphate	TMP
Tri-ethyl phosphate	TEP
Tri-n-propyl phosphate	TPP (TPrP)
Tri-n-butyl phosphate	TNBP
Tris (2-chloroethyl) phosphate	TCEP
Tris (chloroisopropyl) phosphate	TCIPP
Tri-phenyl phosphate	TPHP
2-ethylhexyl diphenyl phosphate	EHDPP
Tri-*m*-tolyl phosphate	m-TMPP (m-TTP)
Tri-*p*-tolyl phosphate	p-TMPP (p-TTP)

Tri-*o*-tolyl phosphate	o-TMPP (o-TTP)
Tris (2-butoxyethyl) phosphate	TBOEP
Tris(3,5-dimethylphenyl) phosphate	T35DMPP
Tris (1,3-dichloro-2-propyl) phosphate	TDCIPP (TDCIPP)
Tris (2-ethyl hexyl) phosphate	TEHP
Tris (2-isopropylphenyl) phosphate	TIPPP (T2IPPP)
Tris (tribromo neopentyl) phosphate	TTBPP
Tris (2,3-dibromopropyl) phosphate	TDBPP
d_{15} tri-ethyl phosphate	d_{15}TEP
d_{21} tri-n-propyl phosphate	d_{21}TPP
d_{27} tri-butyl phosphate	d_{27}TNBP
d_{12} tris (2-chloroethyl) phosphate	d_{12}TCEP
$^{13}c_{18}$ tri-phenyl phosphate	$^{13}C_{18}$ TPHP
$^{13}c_{6}$ tris (2-butoxyethyl) phosphate	$^{13}C_{6}$ TBOEP
d_{15} tris (1,3-dichloro-2-propyl) phosphate	d_{15}TDCIPP
d_{15} tri-phenyl phosphate	*d_{15}TPHP*
Mirex	*Mirex*
2,4,4',6-Tetrabromodiphenyl ether	BDE-75
2,2',4,4'-Tetrabromo[$^{13}C_{12}$]diphenyl ether	MBDE-47
2,2',3,4,4',6-hexabromo[$^{13}C_{12}$]diphenyl ether	MBDE-139
2,2',3,4,4',5,5′-heptabromo[$^{13}C_{12}$]diphenyl ether	MBDE-180
Decabromo[$^{13}C_{12}$]diphenyl ether	MBDE-209
Pentabromo[$^{13}C_{6}$]benzene	MPBBZ
Cl10 Dechlorane plus	aClDP
α-Hexabromo[$^{13}C_{12}$]cyclododecane	Mα-HBCDD
β-Hexabromo[$^{13}C_{12}$]cyclododecane	Mβ-HBCDD
γ-Hexabromo[$^{13}C_{12}$]cyclododecane	Mγ-HBCDD
γ-Hexabromocyclododecane-d18	*Dγ-HBCDD*
Tetrabromo[$^{13}C_{12}$]bisphenol-A	MTBBPA

Internal standards are listed in italicized font.

Table S3. List of genes included on the Avian ToxChip PCR array and their related functions.

Genes	Description	Function
CYP3A37	Cytochrome P450 A 37	Xenobiotic metabolism
CYP1A4	Cytochrome P450 1A4	
UGT1A9	UDP glucuronosyltransferase 1 family, polypeptide A9	
SULT1B1	Sulfotransferase family, cytosolic, 1B, member 1	
NAT	N-acetyltransferase, liver isozyme	
AOC1	Amine oxidase, copper containing 1	
MAT1A	Methionine adenosyltransferase I, alpha	
BATF3	Basic leucine zipper transcription factor, ATF-like 3	Immune function
IL16	Interleukin 16 (lymphocyte chemoattractant factor)	
IL1B	Interleukin 1 Beta	
NOS2	Nitric oxide synthase 2, inducible	
LEAP2	Liver expressed antimicrobial peptide 2	
FGA	Fibrinogen alpha chain	

SULT1E1	Sulfotransferase family 1E, estrogen-preferring member1	Hormone regulation & metabolism
TXN	Thioredoxin	Oxidative stress & inflammation
CA3B	Carbonic anhydrase III-like (LOC420209)	
MGST3	Microsomal glutathione S-transferase 3	
MT4	Metallothionein 4	
ACSL5	Acyl-CoA synthetase long-chain family member 5	Lipid homeostasis
FGF19	Fibroblast growth factor 19	
CYP7B1	Cytochrome P450, family 7, subfamily B, polypeptide 1	
SCD	Stearoyl-CoA desaturase (delta-9-desaturase)	
LSS	Lanosterol synthase (2,3-oxidosqualene-lanosterol cyclase)	
ALDH1A1	Aldehyde dehydrogenase 1 family, member A1	
APOB	Apolipoprotein B	
LBFABP	Fatty acid binding protein 1, liver	
SLCO1A2	Solute carrier organic anion transporter family, member 1A2	
TTR	Transthyretin	Thyroid Hormone pathway
THRSP	Thyroid hormone responsive (SPOT14 homolog, rat)	
IGF1	Insulin-like growth factor 1 (somatomedin C)	
PDK4	Pyruvate dehydrogenase kinase, isozyme 4	Glucose and fatty acid metabolism
G6PC	Glucose-6-phosphatase catalytic subunit (LOC100857298)	
HMOX1	Heme oxygenase 1	Heme homeostasis
ALAS1	Aminolevulinate, delta, synthase1	
CDKN1A	Cdk inhibitor CIP1 (p21)	DNA Damage & Repair
GADD45A	Growth arrest and DNA-damage-inducible, alpha	
MGMT	O-6-methylguanine-DNA methyltransferase	
TP63	Tumor protein p63	
MSH2	MutS homolog 2, colon cancer, nonpolyposis type 1 (E. coli)	
POLB	Polymerase (DNA directed), beta	
POLK	Polymerase (DNA directed) kappa	
FOXA1	Forkhead box A1	Transcriptional regulation
CRYAB	Crystallin, alpha B	Molecular chaperone
EEF1A1	Eukaryotic translation elongation factor 1 alpha 1	Control genes
RPL4	Ribosomal protein L4	
GGDC	Chicken Genomic DNA Contamination	
RTC	Reverse Transcription Control	
PPC	Positive PCR Control	

Table S4. Total concentrations (pg/mL) of organic flame retardants (OFRs), including organophosphate esters (OPEs), polybrominated diphenyl ethers (PBDEs), novel flame retardants (NFRs), hexabromocyclododecanes (HBCDDs) and tetrabromobisphenol A (TBBPA) measured in passive air samplers collected from 19 major cities. The cities are listed in decreasing order based on their total OFR concentrations. Location names are colored based on the United Nations regional groups: Africa (red); Asia-Pacific (green); WEOG = Western Europe and Other states Group (purple); GRULAC = Group of Latin American and the Caribbean (blue); CEE = Central and Eastern Europe (orange).

Location Name	Total OFRs	Σ_{10}OPEs	Σ_{9}PBDEs	Σ_{11}NFRs	ΣHBCDDs/TBBPA
London, UK	4,605, 588	4,564,993	31,638	7408	1550
New York, USA	3,291,515	3,223,693	26,082	34,740	7000
Tokyo, Japan	1,10,6043	1,028,493	19,299	15,,351	42,900
Beijing, China	877,733	825,593	20,092	8523	23,525
Lagos, Nigeria	747,208	647,493	46,993	41572	11,150
Sao Paulo, Brazil	682,286	666,193	6650	8343	1100
Toronto, Canada	65,2355	622,493	12,967	12,770	4125
Bogota, Columbia	473,193	454,893	4030	13,469	800
Bangkok, Thailand	458,288	412,493	23,600	21,321	875
Warsaw, Poland	388,107	381,193	3048	3292	575
Santiago, Chile	322,877	306,565	5039	11,073	200
Mexico City, Mexico	303,891	258,393	12,654	32,269	575
Sydney, Australia	302,907	295,993	1722	3392	1800
Madrid, Spain	297,909	281,593	8022	8294	ND
Istanbul, Turkey	249,617	227,380	5134	1877	15,225
Cairo, Egypt	188,046	158,265	16,784	11,497	1500
New Delhi, India	150,264	127,793	8143	6629	7700
Buenos Aires, Argentina	88,827	83,693	2436	2698	0
Kolkata, India	85,481	75,836	3657	5689	300

Table S5. Raw luminescence cell viability data from CEH dosed with varying concentrations of passive air sampler extracts. Signals were obtained using the ViaLight plus kit and luminescence was read with a 1 s integrated reading time using a luminometer.

Sample Labels	DMSO	0.01	0.1	1
WE01	14.38	16.12	10.95	4.236
WE01	14.38	15.05	12.44	3.85
WE02	14.38	17.16	15.9	14.21
WE02	14.38	14.53	17.19	15.5
WE03	14.38	17.87	17.25	9.64
WE03	14.38	18.45	17.08	10.12
WE05	14.38	16.61	18.08	7.095
WE05	14.38	17.75	15.34	5.116
WE06	14.38	18.4	11.93	4.299
WE06	14.38	18.6	17.38	5.082
GR01	14.38	18.31	18.99	2.755
GR01	14.38	18.38	19.15	3.518
GR02	14.38	20.24	19.35	2.812
GR02	14.38	18.71	19.69	1.583
GR03	14.89	13.15	11.08	7.361
GR03	14.89	14.7	10.35	5.027
GR04	14.89	13.5	12.74	2.813
GR04	14.89	14.34	12.87	2.239
GR05	14.89	15.87	15.51	17.92
GR05	14.89	17.13	16.33	14.66
AS01	14.89	15.16	15.48	8.301
AS01	14.89	16.19	17	7.15
AS02	14.89	17.15	12.6	4.263
AS02	14.89	16.42	17.41	3.641
AS04	14.89	16.88	16.5	5.639
AS04	14.89	17.91	18.3	4.265
AS05	14.89	16.96	15.46	4.51
AS05	14.89	18.71	17.61	4.093
AF01	14.89	13.45	20.9	6.7
AF01	14.89	16.81	17.15	6.267
AF02	14.89	17.53	23.36	3.322
AF02	14.89	18.67	22.52	3.634
CEE01	14.89	15.41	19.27	3.475
CEE01	14.89	17.28	17.84	3.639

Table S6. The ranks of chemicals that contributed to partial least square (PLS) regression modeling for estimating cytotoxicity values (LC50).

Chemicals	Coefficient Values Contributed to PLS Modeling	Ranks
PBEB	0.30	1
BDE-154	0.25	2
BDE-85	0.24	3
syn-DP	0.23	4
TEP	0.20	5
TDCPP	0.19	6
DPTE	0.13	7
anti-DP	0.02	8
BDE-100	0.01	9

Table S7. Raw cycle threshold (Ct) data derived from the Chicken ToxChip PCR array for 43 target genes and four control genes in chicken embryonic hepatocytes exposed to the DMSO vehicle control (A) or a dilution factor of 0.1 from the "neat" air sampler extract from Bangkok, Thailand (B). Average Ct values (± standard deviation) are included (n = 3 replicate wells/treatment group).

A

Symbol	Well	Control Sample				
		**exp1**	**exp2**	**exp3**	**AVG**	**SD**
CYP3A7	A01	21.24	21.03	21.27	21.18	0.13
CYP1A4	A02	24.05	23.59	24.05	23.90	0.27
UGT1A9	A03	24.83	24.67	24.68	24.73	0.09
SULT1B1	A04	24.13	23.6	24.27	24.00	0.35
BATF3	A05	24.98	24.93	25.69	25.20	0.43
PDK4	A06	28.42	27.88	28.39	28.23	0.30
TXN	B01	18.01	17.68	17.8	17.83	0.17
ACSL5	B02	24.06	23.71	24.06	23.94	0.20
SLCO1A2	B03	22.17	21.87	22.31	22.12	0.22
TTR	B04	15.73	15.37	16.03	15.71	0.33
HMOX1	B05	22.3	22.04	22.93	22.42	0.46
THRSP	B06	18.53	17.98	18.22	18.24	0.28
IGF1	C01	30.19	29.06	30.84	30.03	0.90
SULT1E1	C02	21.67	21.22	21.77	21.55	0.29
CYP7B1	C03	25.78	25.26	25.96	25.67	0.36
FGF19	C04	24.33	24.16	24.79	24.43	0.33
ALAS1	C05	19.61	19.24	20.19	19.68	0.48
IL16	C06	27.71	27.5	27.57	27.59	0.11
MT4	D01	23.36	22.71	23.33	23.13	0.37
SCD	D02	17.32	17.16	17.58	17.35	0.21
LBFABP	D03	20.68	20.04	20.85	20.52	0.43
CDKN1A	D04	23.28	23.06	23.37	23.24	0.16
GADD45A	D05	22.15	21.91	22.51	22.19	0.30
MGMT	D06	24.27	23.72	24.08	24.02	0.28
NAT2	E01	24.41	24.07	24.34	24.27	0.18
ALDH1A1	E02	21.08	20.58	21.18	20.95	0.32
MSH2	E03	24.13	23.66	24.21	24.00	0.30
CRYAB	E04	31.99	31.96	32.27	32.07	0.17
FOXA1	E05	23.42	23.26	23.87	23.52	0.32
APOB	E06	19.54	19.18	19.52	19.41	0.20
POLB	F01	26.11	25.7	26.04	25.95	0.22
POLK	F02	26.39	26.02	26.59	26.33	0.29
TP63	F03	29.87	29.47	29.81	29.72	0.22
G6PC	F04	20.56	20.39	20.56	20.50	0.10
CA3B	F05	29.77	28.87	30.37	29.67	0.75
LSS	F06	22.7	22.26	22.57	22.51	0.23
AOC1	G01	29.32	28.92	28.92	29.05	0.23
MGST3	G02	19.65	19.14	19.55	19.45	0.27
LEAP2	G03	21.64	21.1	21.67	21.47	0.32
FGA	G04	18.47	No Ct	18.74	24.07	9.47
MAT1A	G05	21.43	21.16	21.85	21.48	0.35
IL1B	G06	30.34	30.05	30.23	30.21	0.15
NOS2	H01	27.3	27.43	26.96	27.23	0.24
EEF1A1	H02	16.93	16.44	17.18	16.85	0.38
RPL4	H03	17.76	17.14	17.87	17.59	0.39
GGDC	H04	34.93	**35.63**	34.45	34.79	0.30
PPC	H06	19.33	19.91	19.45	19.56	0.31

B

Symbol	Well	Test Sample				
		**exp1**	**exp2**	**exp3**	**AVG**	**SD**

CYP3A7	A01	21.32	21.26	21.25	21.28	0.04
CYP1A4	A02	23.55	23.15	23.11	23.27	0.24
UGT1A9	A03	24.36	24.36	24.03	24.25	0.19
SULT1B1	A04	23.55	23.17	23.09	23.27	0.25
BATF3	A05	24.99	26.27	25.19	25.48	0.69
PDK4	A06	27.85	27.47	27.69	27.67	0.19
TXN	B01	17.58	17.31	17.06	17.32	0.26
ACSL5	B02	23.34	23.29	23.35	23.33	0.03
SLCO1A2	B03	21.75	21.79	21.63	21.72	0.08
TTR	B04	15.43	15.56	15.26	15.42	0.15
HMOX1	B05	21.53	22.39	21.39	21.77	0.54
THRSP	B06	18.63	18.39	18.51	18.51	0.12
IGF1	C01	28.76	29.11	28.94	28.94	0.18
SULT1E1	C02	21.03	20.9	20.96	20.96	0.07
CYP7B1	C03	25.06	24.6	24.72	24.79	0.24
FGF19	C04	24.07	24.11	23.93	24.04	0.09
ALAS1	C05	18.66	19.42	18.44	18.84	0.51
IL16	C06	27.88	27.57	27.53	27.66	0.19
MT4	D01	23.44	24.8	23.24	23.83	0.85
SCD	D02	17.3	17.34	17.27	17.30	0.04
LBFABP	D03	20.26	20.4	20.29	20.32	0.07
CDKN1A	D04	23.4	23.42	23.33	23.38	0.05
GADD45A	D05	21.99	22.14	21.6	21.91	0.28
MGMT	D06	24.01	23.5	23.74	23.75	0.26
NAT2	E01	23.98	24.05	23.93	23.99	0.06
ALDH1A1	E02	20.24	20.1	20.29	20.21	0.10
MSH2	E03	23.64	23.79	23.54	23.66	0.13
CRYAB	E04	31.97	31.53	31.55	31.68	0.25
FOXA1	E05	23.03	23.12	22.97	23.04	0.08
APOB	E06	18.91	18.53	18.85	18.76	0.20
POLB	F01	25.19	25.46	25.36	25.34	0.14
POLK	F02	26.05	25.44	25.46	25.65	0.35
TP63	F03	30.09	29.93	30.23	30.08	0.15
G6PC	F04	20.46	20.87	20.55	20.63	0.22
CA3B	F05	28.98	29.49	29.9	29.46	0.46
LSS	F06	22.53	22.25	22.43	22.40	0.14
AOC1	G01	29.07	28.81	28.61	28.83	0.23
MGST3	G02	19.08	18.96	18.96	19.00	0.07
LEAP2	G03	21.61	21.46	21.21	21.43	0.20
FGA	G04	18.3	18.61	18	18.30	0.31
MAT1A	G05	21.06	21.08	21.06	21.07	0.01
IL1B	G06	29.8	29.68	29.66	29.71	0.08
NOS2	H01	26.92	26.94	26.65	26.84	0.16
EEF1A1	H02	16.48	16.27	16.48	16.41	0.12
RPL4	H03	17.61	17.7	17.47	17.59	0.12
GGDC	H04	No Ct	No Ct	No Ct	**35.00**	0.00
PPC	H06	19.63	18.9	18.72	19.08	0.48

Table S8. Fold changes calculated from the Chicken ToxChip PCR array. Target genes were considered significantly dysregulated if FDR adjusted t-test p-value < 0.05 and 0.5 < fold change > 2 (indicated in orange).

Genes	DMSO	Toronto, Canada	New York, USA	Sydney, Australia	Istanbul, Turkey	London, UK	Kolkata, India	Beijing, China	Bangkok, Thailand	Mexico City, Mexico	Santiago, Chile	Buenos Aires, Argentina	Lagos, Nigeria	Cairo, Egypt
**ACSL5**	0.00	-1.74	-0.39	-0.71	0.28	-0.68	0.26	-0.49	0.68	-0.81	-0.20	-0.04	-0.15	-0.46
**ALAS1**	0.00	1.24	0.64	0.97	1.55	1.35	1.56	1.67	0.49	1.75	1.23	0.53	1.87	1.62
**ALDH1A1**	0.00	-0.39	0.23	0.35	0.60	0.27	1.07	0.65	0.58	0.42	0.83	0.35	1.06	0.65
**AOC1**	0.00	-1.15	-0.28	-0.90	-0.14	-0.37	-0.72	-1.56	0.51	-1.25	-0.94	-0.35	-1.04	-1.37
**APOB**	0.00	-2.43	-0.73	-0.97	-0.24	-1.22	-1.11	-1.67	0.93	-1.73	-1.27	-1.00	-1.31	-1.32
**BATF3**	0.00	-0.46	0.05	0.24	-0.62	-0.02	0.18	-0.03	-0.61	-0.16	0.12	0.43	-0.11	-0.18
**CA3B**	0.00	-0.93	-0.13	-0.24	-0.75	-0.75	-0.77	-2.21	-0.07	-1.38	-0.44	-0.15	-0.87	-1.03
**CDKN1A**	0.00	-0.84	-0.53	-0.61	-0.99	-0.69	-0.77	-0.71	-0.40	-0.49	-0.54	-0.67	-0.70	-0.88
**CRYAB**	0.00	-0.37	0.70	-1.00	0.52	0.63	-0.04	-0.49	0.53	-0.85	-1.31	-0.56	-1.50	-1.09
**CYP1A4**	0.00	1.64	1.71	1.86	-0.01	1.56	2.66	1.61	0.69	1.97	1.59	1.29	4.44	1.43
**CYP3A7**	0.00	-1.50	-1.03	-0.67	-0.89	-0.73	-0.99	-1.58	-0.12	-1.41	-0.59	-0.32	-0.75	-1.36
**CYP7B1**	0.00	-2.81	-0.10	-0.55	-0.78	-1.08	0.00	-1.01	0.78	-1.49	-0.22	-0.33	-0.59	-0.88
**FGA**	0.00	-4.48	-0.65	-1.15	-0.39	-2.09	-0.55	-2.09	1.13	-2.62	-0.47	0.62	-1.44	-1.77
**FGF19**	0.00	2.45	-0.27	0.27	1.70	1.39	0.60	2.06	-0.02	1.61	0.20	-0.95	-0.26	1.59
**FOXA1**	0.00	-1.68	-0.55	-0.82	0.21	-0.96	-0.60	-1.11	0.66	-0.81	-0.75	-0.73	-0.91	-1.29
**G6PC**	0.00	-3.52	-1.29	-1.71	-0.25	-1.99	-1.03	-1.39	0.13	-1.86	-1.59	-1.29	-1.59	-2.04
**GADD45A**	0.00	-0.21	-0.31	-0.33	0.50	-0.07	0.11	0.08	0.15	0.03	0.02	-0.09	-0.06	0.01
**HMOX1**	0.00	0.72	0.92	0.99	0.37	1.18	1.46	1.62	0.19	1.61	1.19	0.72	1.73	1.65
**IGF1**	0.00	-2.40	0.37	0.32	-0.87	-1.96	-0.42	-1.50	0.91	-2.14	0.65	0.86	-1.12	-1.19
**IL16**	0.00	-0.84	-1.16	-1.60	-0.61	-1.63	-1.39	-1.81	0.04	-1.54	-1.82	-1.26	-1.67	-1.58
**IL1B**	0.00	-0.06	-0.04	-0.02	-0.08	-0.53	-1.71	-0.89	0.27	-0.94	-0.72	-1.35	-0.75	-1.18
**LBFABP**	0.00	-1.95	-0.03	0.04	-0.33	-0.86	-0.19	-0.68	0.04	-1.15	-0.12	0.24	-0.76	-1.20
**LEAP2**	0.00	-2.87	-0.69	-0.87	-0.19	-1.07	-0.03	-0.60	0.20	-1.21	-0.56	-0.62	-0.78	-1.13
**LSS**	0.00	-2.45	-1.22	-1.32	-1.03	-1.71	-1.33	-1.86	0.27	-1.67	-1.09	-0.61	-1.46	-1.59
**MAT1A**	0.00	-1.23	-0.42	-0.60	-0.39	-0.94	-0.30	-0.96	0.37	-0.71	-0.13	0.30	-0.52	-0.71
**MGMT**	0.00	-0.21	0.12	0.10	-0.20	0.09	-0.04	0.00	0.07	0.00	0.08	0.08	0.04	-0.07
**MGST3**	0.00	0.25	0.20	0.49	0.90	0.47	0.69	0.76	0.24	0.81	0.67	0.14	0.68	0.59
**MSH2**	0.00	-0.23	-0.20	-0.26	-0.02	-0.29	0.02	-0.13	0.17	-0.03	-0.04	0.09	-0.01	-0.04
**MT4**	0.00	-0.19	0.44	0.34	-0.64	0.18	-0.24	-0.38	-1.03	0.12	-0.65	0.32	-0.73	-0.46
**NAT2**	0.00	-1.05	-0.32	-0.31	0.47	-0.26	-0.64	-0.17	0.27	-0.38	-0.15	-0.37	-0.13	-0.32
**NOS2**	0.00	-0.21	-0.28	-0.12	-0.41	-0.33	-0.22	-0.42	-0.06	-0.36	-0.28	-0.02	-0.26	-0.33
**PDK4**	0.00	1.85	-0.09	0.08	1.19	1.20	1.37	1.58	0.65	1.26	0.73	-0.50	1.26	1.74
**POLB**	0.00	-0.32	-0.67	-0.58	0.51	-0.59	-0.87	-0.71	0.65	-0.51	-0.99	-0.95	-0.81	-0.70
**POLK**	0.00	-0.65	-0.22	-0.31	0.09	-0.31	-0.16	-0.32	0.63	-0.09	-0.08	-0.10	-0.22	-0.16
**SCD**	0.00	-2.83	-1.20	-1.69	-1.00	-2.01	-1.69	-2.76	0.39	-2.34	-1.47	-1.08	-1.88	-2.42
**SLCO1A2**	0.00	-2.32	-0.16	-0.40	-0.14	-0.69	-0.04	-1.01	0.45	-1.28	-0.06	0.27	-0.52	-1.03

**SULT1B1**	0.00	0.91	0.63	0.49	0.36	0.94	0.85	0.56	0.43	0.51	0.60	0.43	0.61	0.51
**SULT1E1**	0.00	0.84	0.34	0.51	1.13	0.87	1.29	1.52	0.47	1.40	1.03	0.25	1.26	1.19
**THRSP**	0.00	-3.89	-1.25	-1.99	-1.71	-2.79	-1.44	-2.53	-0.34	-2.79	-1.83	-0.96	-2.38	-2.71
**TP63**	0.00	-1.47	-1.52	-1.36	-0.52	-1.65	-1.43	-1.81	-0.34	-1.71	-2.24	-1.38	-1.89	-1.12
**TTR**	0.00	-1.98	-0.08	-0.41	-0.42	-0.68	-0.05	-0.45	0.15	-0.67	0.04	0.29	-0.28	-0.67
**TXN**	0.00	1.45	0.22	0.69	1.34	1.08	1.33	1.89	0.27	1.69	1.03	0.28	1.16	1.42
**UGT1A9**	0.00	-1.55	-1.90	-2.02	0.52	-2.17	-1.63	-1.33	0.49	-1.33	-2.24	-2.25	-1.87	-1.66

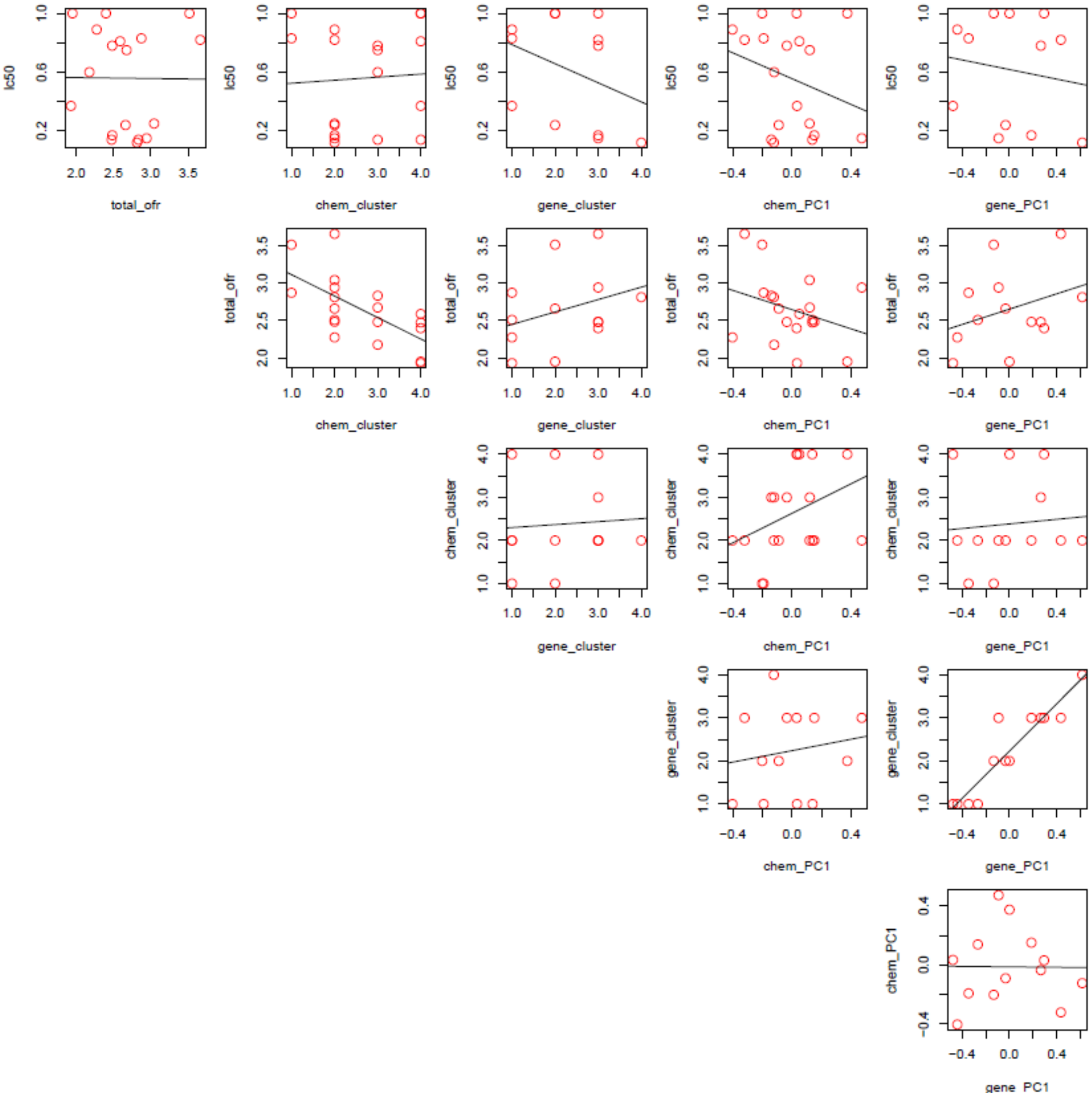

Figure S1. Simple linear regression plots between each of the OFR concentrations, the LC50 values, heatmap clusters and principle component one (PC1) values from chemical burdens and gene expression (detailed in Table 1).

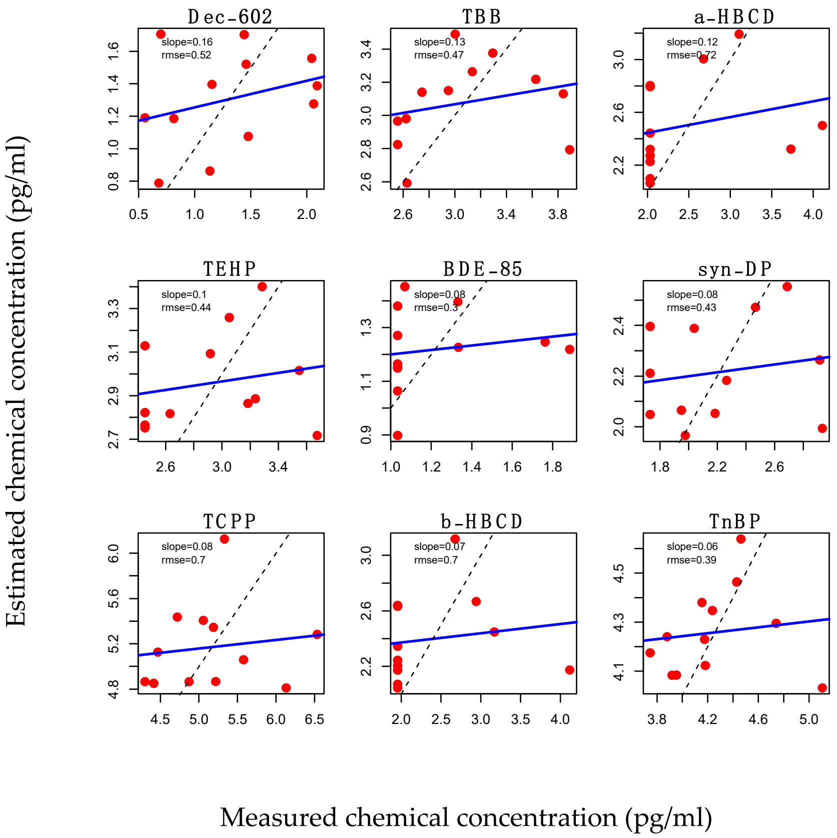

Figure S2. Linear regression plots comparing measured and estimated concentrations of the top nine performing OFRs identified by twelve megacities-based PLS analysis using the gene expression data. 7 of 19 cities were removed: three cities [Tokyo, New Delhi, and Warsaw] failed for RNA extraction, three cities [Sao Paulo, Bogota and Madrid] had unstable expression of housekeeping gene and one outlier [Lagos] had an unusual OFR to bioactivity relationship. The blue line indicates the regression between estimated and actual LC50 values. The dashed line represents a perfect 1-to-1 prediction. Red dots represent air samples collected megacities.

References

1. Saini, A.; Harner, T.; Chinnadhurai, S.; Schuster, J.K.; Yates, A.; Sweetman, A.; Aristizabal-Zuluaga, B.H.; Jiménez, B.; Manzano, C.A.Gaga, E.O. *GAPS-megacities: A new global platform for investigating persistent organic pollutants and chemicals of emerging concern in urban air.* Environmental Pollution, 2020. **267**: p. 115416.
